# TK1 in gastric cancer: Helicobacter pylori-driven oncogenesis biomarker, utility, and emerging targeted therapies

**DOI:** 10.3389/fonc.2026.1641930

**Published:** 2026-03-02

**Authors:** Zi-xuan Zheng, Lu Yang, Jun-tong Chen, Yan Zeng, Shi-xue Dai, Shi-jie Liu

**Affiliations:** 1 Department of Gastroenterology, Geriatric Center, National Regional Medical Center, Guangdong Provincial People’s Hospital Ganzhou Hospital, Ganzhou Municipal Hospital, Ganzhou, Jiangxi, China; 2 The Second School of Clinical Medicine, Southern Medical University, Guangzhou, Guangdong, China; 3 Department of Gastroenterology, Guangdong Provincial Geriatrics Institute, National Key Clinical Specialty, Guangdong Provincial People's Hospital, Guangdong Academy of Medical Sciences, Southern Medical University, Guangzhou, Guangdong, China

**Keywords:** gastric cancer, Helicobacter pylori, serum biomarker, targeted therapies, thymidine kinases

## Abstract

As a leading cause of cancer-related mortality among gastrointestinal malignancies, gastric carcinoma (GC) necessitates enhanced molecular diagnostic paradigms for early intervention. The thymidine kinase enzymatic family, particularly thymidine kinase 1 (TK1, EC 2.7.1.21), serves as an enzymatic orchestrator of deoxyribonucleotide salvage pathways critical for genomic integrity maintenance, and its oncogenic overexpression acts in concert with *Helicobacter pylori*-mediated chronic inflammatory microenvironments, and potentiates the histopathological progression from premalignant metaplasia review delineates the utility of TK1 as a serological biomarker for early detection, tumor staging, therapeutic monitoring, and prognostic stratification in GC. We have hypothesized the molecular mechanisms underlying TK1-mediated oncogenesis and its interplay with *H. pylori*-induced pathogenesis. Additionally, we have explored emerging TK1-targeted therapeutic modalities, including gene-directed enzyme prodrug therapy (GDEPT), nanoscale drug delivery platforms, and adoptive cell therapy, while evaluating TK1’s translational potential in GC management. Although the precise regulatory networks of TK1 in gastric carcinogenesis remain incompletely characterized, ongoing research positions TK1 as a promising diagnostic and therapeutic target, potentially revolutionizing strategies to ameliorate clinical outcomes.

## 1 Introduction

Gastric carcinoma (GC) is a leading cause of cancer-related mortality and a global health burden. Due to its frequently advanced stage at diagnosis, mortality is high. GC is the third most common cause of cancer-related deaths, with 9.7 million deaths globally in 2022 (1). Carcinogenesis involves multifactorial interactions, with *Helicobacter pylori (H. pylori)* infection, genetic susceptibility, and environmental triggers acting synergistically (2–4). Due to the asymptomatic nature of early-stage GC, over 90% of cases are diagnosed at advanced phases, correlating with a dismal 5-year survival rate of ~5% (5). GC progression usually involves a premalignant phase, marked by dysregulated cellular proliferation. TK1, a rate-limiting enzyme in the pyrimidine salvage pathway, is essential for DNA replication and genomic stability (6). High TK1 expression signals increased cell proliferation and may indicate tumor growth (7). Conventional chemotherapeutics, such as 5-fluorouracil, remain the standard of GC, but resistance reduces their efficacy (8). New TK1-centric therapies—including gene editing, nanoparticle drug delivery, and engineered cellular interventions—offer greater specificity and reduced off-target toxicity, signaling a shift in GC treatment.

Significant strides have been made in uncovering the biological role and clinical significance of TK1 in gastric cancer. Yet, the intricacies of its molecular regulatory network and its potential translational applications demand urgent exploration. On one hand, the specific signaling pathways through which TK1 drives gastric carcinogenesis remain shrouded in mystery. On the other hand, although a correlation exists between Helicobacter pylori (H. pylori) infection and TK1 dysregulation, we still lack compelling experimental evidence to confirm whether a direct synergistic carcinogenic mechanism arises from H. pylori infection or whether it is an indirect effect mediated by inflammation.Addressing these gaps is essential for advancing our understanding and treatment of this formidable disease.

This review synthesizes insights into TK1’s regulatory roles in GC progression, emphasizing its dual utility as a diagnostic biomarker and therapeutic target, and elucidates the molecular mechanisms and therapeutic potential of TK1 in H. pylori-associated gastric cancer. We further dissect the mechanistic crosstalk between *H. pylori* infection and TK1 dysregulation, while critically appraising innovative therapeutic strategies to inform future research and clinical translation.

## 2 Thymidine kinase family: from structure to function

### 2.1 TK1: a proliferation-driven enzyme

Thymidine kinase (TK) is a key enzyme in DNA synthesis and repair, providing the raw material for DNA replication by phosphorylating deoxythymidine to deoxythymidylate (dTMP). The thymidine kinase family contains multiple isoenzymes whose functions vary according to subtype and cellular localization (**Table 1**).

**Table 1:** TK and its isoenzymes.

Isoenzyme type	Localization	Primary function	Key characteristics	Implications for therapy
TK1	Cytoplasm	Catalyzes phosphorylation of deoxythymidine to dTMP for DNA synthesis and repair.	Cell cycle-dependent (high in S phase); elevated in malignant tumors (e.g., liver, breast, lung cancer); used for diagnosis and prognosis.	Target for GDEPT (e.g., GCV); predictive biomarker for therapy response
TK2	Mitochondria	Maintains mitochondrial DNA (mtDNA) stability and regulates pyrimidine nucleotide metabolism.	Cell cycle-independent; mutations linked to mitochondrial diseases (e.g., myopathy, encephalomyopathy); supports energy production.	Not a primary target in GC; potential role in mitigating therapy-induced toxicity
Viral TK	Viral particles	Facilitates viral DNA replication; target for antiviral drugs.	Encoded by viruses (e.g., herpes simplex virus); structural similarity to host TK but different substrate specificity; inhibits viral replication.	Key target for antiviral drugs (e.g., acyclovir);tool for suicide gene therapy (HSV-TK/GCV) in GC
Bacterial TK	Bacterial cells	Supports bacterial growth, survival, and adaptation to environmental stress.	Essential for growth and reproduction; mutations cause slow growth or reduced survival; helps in DNA damage response.	Potential target for novel anti-Hp antibiotics

dTMP, deoxythymidine monophosphate; mtDNA, mitochondrial DNA; GDEPT, Gene-Directed Enzyme Prodrug Therapy; GCV, ganciclovir; HSV-TK, Herpes Simplex Virus Thymidine Kinase;, GC, gastric cancer.

TK1 is located in the cytoplasm and catalyzes nuclear DNA proliferation, TK1 is cell cycle-dependent and elevated in cancers, serving as a diagnostic and prognostic biomarker. TK2 is in the mitochondria and maintains mtDNA stability. Viral TK can participate in the replication of genetic information in DNA viruses and serves as a target for antiviral therapy and suicide gene therapy approaches. Bacterial TK promotes bacterial DNA synthesis, affecting bacterial reproduction and resistance to external stress. Bacterial TK represents a potential target for novel antibiotics.

Building on this, the main isoenzymes of TK include TK1, TK2, viral thymidine kinase, and bacterial thymidine kinase. Each participates in different aspects of thymidine metabolism, including phosphorylation and repair pathways.

Among the TK isoenzymes, TK1 is of particular interest in cancer biology due to its cell-cycle-dependent cytoplasmic expression. Its activity peaks during the S-phase, making it a sensitive marker of cellular proliferation (9). TK1 is highly expressed in various malignant tumors (e.g., liver, breast, and lung cancer), and its serum levels are positively correlated with tumor proliferation, making it useful for early diagnosis, efficacy assessment, and prognostic evaluation (10, 11).

### 2.2 Mitochondrial TK2 and microbial TKs

TK2 is mainly found in the mitochondria, and its expression is independent of the cell cycle. It is primarily responsible for maintaining mitochondrial DNA (mtDNA) stability. Mutations in TK2 are associated with mitochondrial diseases (e.g., myopathy, encephalomyopathy) because they affect the repair and replication of mtDNA. Deficiency in TK2 function can lead to mtDNA synthesis disorders, including hereditary mitochondrial encephalomyopathies (12). TTK2 can also regulate cellular metabolism by modulating pyrimidine nucleotide metabolism, providing more substrates for mitochondrial energy production to meet increased cellular energy demand.

Viral TK, encoded by certain viruses (e.g., herpes simplex virus and cytomegalovirus), shares structural similarities with host TK but differs in substrate specificity. These enzymes play a key role in viral DNA replication and serve as targets for antiviral drugs. For example, when constructing recombinant Marek’s disease virus (MDV) vaccine strains, deleting the viral TK gene can further attenuate the virus and reduce the lymphoid organ atrophy it causes (13).

Bacterial TK is related to bacterial growth and survival. In microorganisms such as Pseudomonas, thymidine kinase is essential for bacterial growth, reproduction, and survival. Lacking or mutation of this enzyme can lead to phenotypes such as slow growth and reduced survival capabilities (14). It may also play a regulatory role in bacterial adaptation to environmental stresses (e.g., nutrient limitation or DNA damage), enabling bacteria to survive under different conditions.

## 3 TK1 in gastric cancer: pathogenic mechanism and clinical implications

### 3.1 Risk factors for gastric cancer

#### 3.1.1 Helicobacter pylori Infection

*H. pylori* is a microorganism capable of surviving in the acidic environment of the stomach and is one of the major risk factors for gastric cancer. There are two main mechanisms of *H. pylori*’s impact on tumorigenesis: indirect inflammatory responses on the gastric mucosa and direct epigenetic effects on gastric epithelial cells caused by Helicobacter pylori infection (15). Several virulence factors of *H. pylori*, such as CagA or VacA, increase the risk of gastric cancer (GC). The expression of CagA and VacA in *H. pylori* is associated with a stronger tissue reaction and a higher risk of precancerous and malignant lesions in the distal stomach (16, 17). Numerous epidemiological studies have shown that *H. pylori* infection is a risk factor for the development of GC. Additionally, *H. pylori* infection can damage the gastric tissue microenvironment, promote epithelial-mesenchymal transition (EMT), and further the progression of GC (18).

#### 3.1.2 Genetic factors

Genetic factors play a significant role in the development of gastric cancer. Many studies have revealed a clear family history of gastric cancer (19). However, in countries with increased disease incidence, environmental factors have a greater impact on the development of familial GC than genetic changes.

#### 3.1.3 Environmental factors

Long-term intake of high-salt, smoked, and cured foods, as well as foods containing nitrites, can increase gastric cancer risk (20). Long-term smoking and alcohol use are also significant risk factors, with smokers having a 50% higher risk of gastric cancer. Additionally, prolonged exposure to chemicals like asbestos and nickel can increase the risk of gastric cancer.

#### 3.1.4 Precancerous lesions

Gastric polyps, chronic atrophic gastritis, and the remnant stomach post-gastrectomy may involve chronic inflammation, intestinal metaplasia, or atypical hyperplasia of the gastric mucosa, potentially leading to cancer (21).

#### 3.1.5 Immune evasion

Cancer cells transfer mitochondria with mtDNA mutations to tumor-infiltrating lymphocytes (TILs), resulting in T cell metabolic dysfunction and loss of function, which weakens anti-tumor immune responses and promotes the progression of gastric cancer (22).

### 3.2 TK1: the key subtype associated with gastric cancer

Total TK activity increases compared to normal gastric mucosa, with higher activity in poorly differentiated adenocarcinomas than in well-differentiated ones (23). TK1, closely linked to gastric cancer, is significant for diagnosis, therapeutic monitoring, and prognostic assessment. Serum TK1 levels correlate with clinical stage, ECOG PS, and CEA levels in patients with gastric cancer. Dynamic changes in serum TK1 two months before chemotherapy are more crucial than baseline levels for assessing chemotherapeutic response and predicting PFS and RFS (24).

### 3.3 Interaction mechanisms between *H. pylori* infection and thymidine kinase

#### 3.3.1 *H. pylori* Infection drives cell proliferation and upregulates TK1 activity

*H. pylori* infection indirectly affects thymidine kinase (TK1) activity by inducing abnormal proliferation of gastric mucosal cells and disrupting DNA synthesis. As a rate-limiting enzyme in DNA synthesis, TK1 activity is closely linked to cell proliferation, bacterial growth, and the host microenvironment. Clinical studies have shown significantly elevated serum TK1 levels in patients with *H. pylori*-associated precancerous lesions (lung, esophagus, gastric, head and neck, and thyroid), likely reflecting compensatory proliferation following gastric mucosal injury (25).

#### 3.3.2 Dysregulation of cell cycle control and functional links to TK1

*H. pylori* infection promotes uncontrolled proliferation of gastric epithelial cells by disrupting cell cycle checkpoints. Key mechanisms include:

Activation of Cyclin-Dependent Pathways: CagA-positive strains activate the Wnt/β-catenin signaling pathway, upregulate cyclins (e.g., Cyclin D1, Cyclin E) and drive cells into the S phase (26, 27). Since TK1 is a marker enzyme of the S phase, its activity may be synchronously upregulated during this process. Abnormal regulation of the cell cycle directly affects TK1 expression, as TK1 plays a crucial role in DNA synthesis and cell cycle progression (28). Therefore, H. pylori infection upregulates TK1 expression by affecting the expression of cell cycle proteins and the function of cell cycle checkpoint proteins, thereby accelerating cell proliferation and potentially promoting tumor development.

Interference with Inflammatory Signaling: *H. pylori* infection transcriptionally activates SOD2 via the NF-κB pathway, contributing to 5-FU resistance (29, 30), suggesting a synergistic role for TK1 in bacterial-host metabolic crosstalk. On the other hand, research has shown that activation of the PI3K/Akt pathway can enhance TK1 activity, promote cell proliferation and survival, and provide favorable conditions for the development of H. pylori-related tumors (31). In summary, *H. pylori* infection significantly regulates the expression of TK1 through the interaction of multiple signaling pathways, thereby affecting cell proliferation and tumorigenesis.

#### 3.3.3 DNA damage response and epigenetic regulation

The chronic inflammatory microenvironment induced by *H. pylori* infection indirectly modulates TK1-related pathways. Bacterial toxins impair the DNA damage response system, exacerbating genetic mutations and carcinogenesis (32). This replication stress may activate TK1-mediated nucleotide salvage synthesis. Besides, *H. pylori* infection upregulates DNA methyltransferase (DNMT) activity in gastric epithelial cells, leading to hypermethylation of tumor suppressor gene promoters (e.g., p16) (33). Although direct evidence of TK1 gene regulation is lacking, epigenetic modifications of proliferation-related genes may alter TK1 function via metabolic remodeling.

In summary, *H. pylori* drives TK1 dysregulation through both genotoxic (DNA damage) and epigenetic mechanisms, creating a permissive environment for gastric carcinogenesis. Future investigations should directly examine the epigenetic regulation of the TK1 gene itself during infection and explore the potential of combining TK1 activity monitoring with demethylating agents as a novel strategy to intercept the progression from *H. pylori*-induced inflammation to gastric cancer.

#### 3.3.4 Potential role of TK1 in *H. pylori* antibiotic resistance

Drug-resistant *H. pylori* strains pose significant challenges in treating peptic ulcers and gastric cancer (34). Emerging evidence suggests:

TK-Related Gene Mutations and Resistance: Mutations in herpes simplex virus (HSV) thymidine kinase (TK) genes confer acyclovir resistance (35–37), while zidovudine resistance in *Escherichia coli* correlates with TK inactivation (38), implying that altered TK activity may influence antibiotic susceptibility.

**Figure 1:**
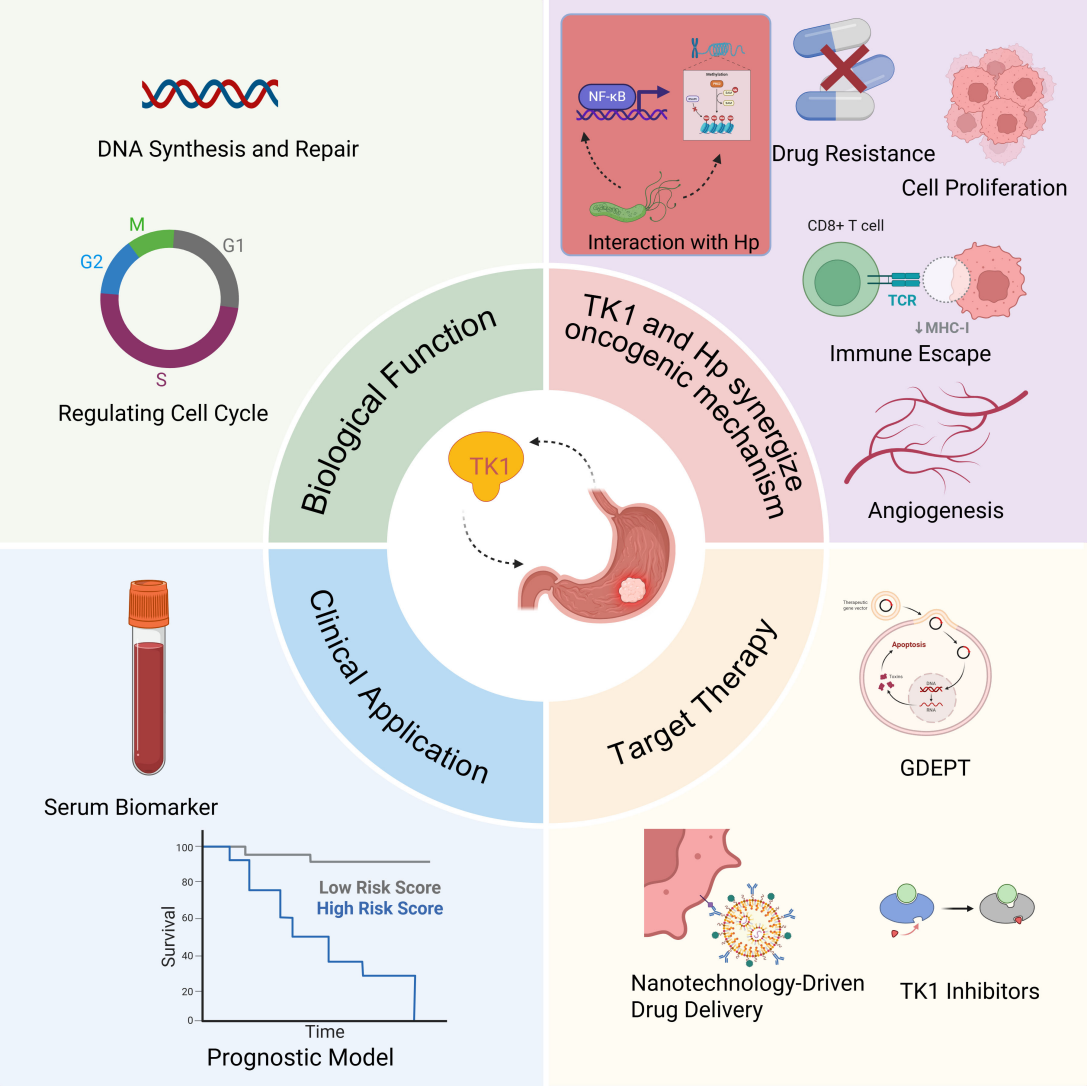
Graphical abstract (Created in BioRender.com). The biological functions of Tk1, the mechanism that causes gastric cancer, its clinical applications, and the development of target therapy based on Tk1. Among them, the interaction with HP is the innovation point of this paper. GDEPT: Gene-Directed Enzyme Prodrug Therapy.

Novel Hypothesis for *H. pylori* Resistance: We hypothesize that *H. pylori* may enhance TK1 enzymatic stability by forming TK1-bacterial complexes or accelerate cell cycle progression through non-canonical pathways, thereby evading antibiotic eradication (**Figure 1**). Targeting TK1 activity regulation could offer novel strategies to overcome resistance.

Future research should focus more on the functional characteristics of TK1 across different stages of H. pylori infection and on its molecular regulatory network. At the same time, researchers should strive to reconcile differences across studies to develop a comprehensive understanding of TK1 in the pathogenesis of gastric diseases.

### 3.4 TK1-mediated cell cycle and metabolic reprogramming

**Figure 2:**
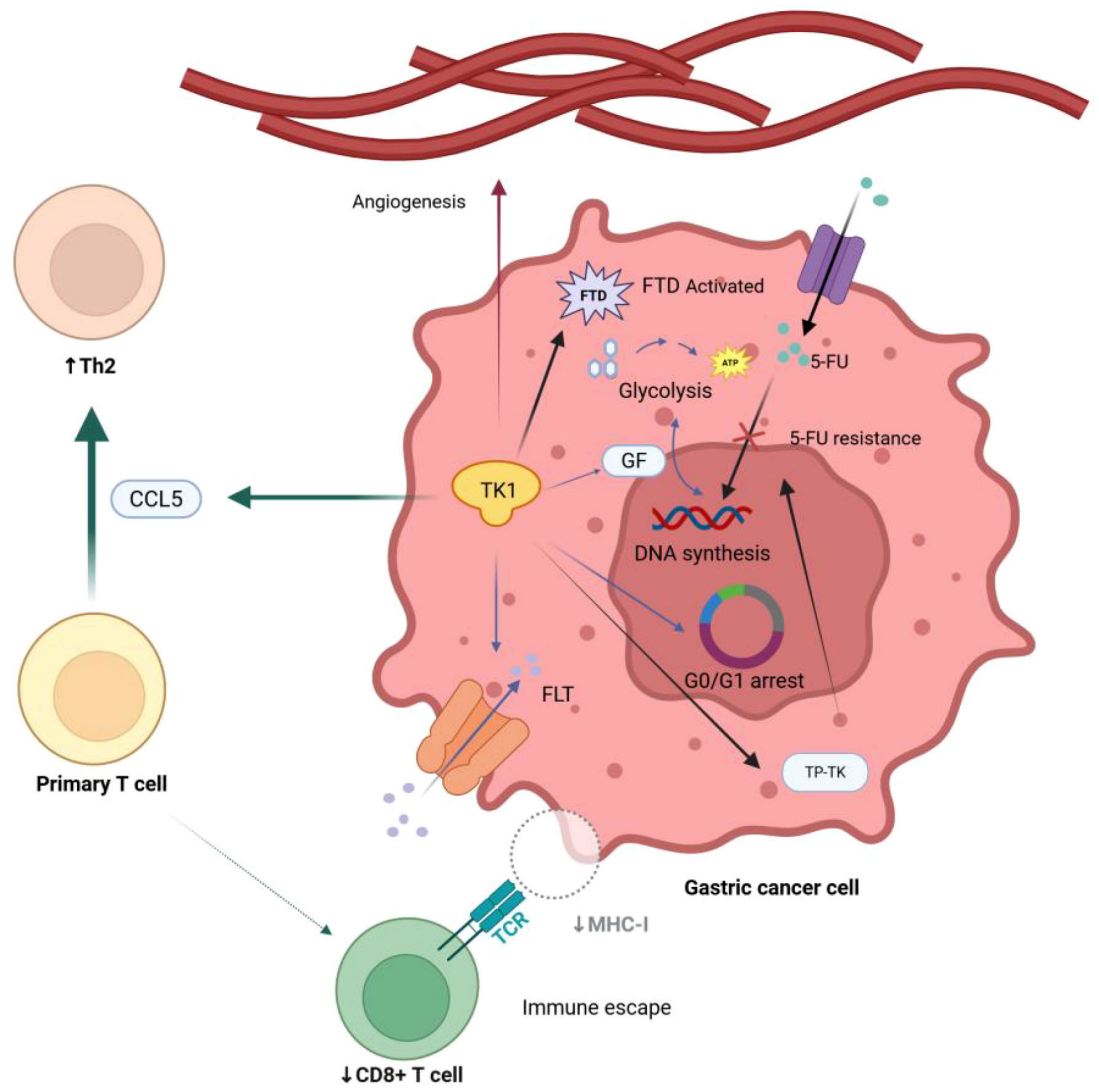
The mechanism by which TK1 may be associated with the occurrence and progression of gastric cancer (Created in BioRender.com). FLT, fluorothymidine; GF, growth factor; FTD, trifluridine; 5-FU, fluorouracil. TK1 promotes serum FLT uptake, which provide materials for DNA synthesis.; TK1 catalyzes GF-mediated glycolysis and DNA synthesis; TK1 decreases G0/G1 arrest period, promote the mitosis of cancer cells.; TK1 regulates Th2 cell polarization through the chemokine CCL5, resulting in great reduction of CD8+T cells, mediating immune escape of cancer cells; TK1 promotes peritumoral angiogenesis, playing an important role in regulating tumor dormancy, proliferation, and influencing the tumor immune microenvironment; TK1 leads to 5-FU resistance through TP-TK pathway and activates FTD, leading to drug resistance of cancer cells.

RNA sequencing shows dramatic upregulation of TK1-related gene expression in neuroblastoma cells (39), indicating that high TK1 expression is positively correlated with cancer progression and invasion (**Figure 2**). Therefore, a pan-cancer analysis suggests that TK1 is not only an essential biomarker for tumor occurrence and development but may also be a potential target for future treatment (25).

#### 3.4.1 TK1 promote DNA synthesis and cancer cell proliferation

TK1 drives DNA salvage synthesis, critically supporting proliferation in differentiated GC (40). In addition, TK1 activity is a vital determinant of FLT uptake in gastrointestinal cancer (41). Growth factors(GF) promote tumor development through TK1-mediated DNA synthesis and glycolysis (42). Through a study on adrenocortical carcinoma, we can infer that the overexpression of TK1 not only serves as a byproduct of the cancer process but also as a crucial part of the selection process, helping cancer cells progress (43).

#### 3.4.2 TK1-mediated immune evasion

Research has indicated that Th2 cells display the closest correlation with TK1 among all kinds of immune cells in Pan-cancer. *In vitro* experiments showed that TK1 affects cancer cell proliferation by regulating G0/G1 phase arrest. Besides, TK1 in cancer cells was also found to regulate Th2 cell polarization through the chemokine CCL5 (44), which is closely associated with gastric cancer cells immune escape since its high expression is a potential indicator for immune evasion of renal cell carcinoma (45).

#### 3.4.3 TK1 leads to drug resistance

TK1 is also associated with resistance of gastric cancer cells to 5-fluorouracil(5-FU). Mori et al. found that FdUMP triggers 5-FU resistance by reducing the thymidine phosphorylase-thymidine kinase (TP-TK) pathway, blocking 5-FU incorporation into nucleic acids, and that *TK* overexpression may play a significant role in the 5-FU resistance of these cells (46). TK is a key enzyme that activates FTD in cancer cells; cells lacking TK activity are less sensitive to FTD (47).

#### 3.4.4 Speculative associations between TK1 and tumor microenvironment

A study on lung cancer has shown that TK1 could promote peritumoral angiogenesis and provide a nutrient and metabolic basis for the growth of tumor cells (48). Although there is currently no research to confirm this, many have speculated that TK1 may be associated with T cell exhaustion and macrophage polarization.In the tumor microenvironment, the dynamic regulation of local oxidative stress is closely related to the metabolic reprogramming of cancer cells. *H. pylori* binds to free heme released from hemoglobin, inducing an increase in reactive oxygen species (ROS) levels, and in turn activates oxidative stress-related signaling pathways such as nuclear factor kappa B (NF-κB) (49–51). Emerging evidence indicates that this oxidative-stress microenvironment can significantly upregulate TK1 expression (52). The activation of TK1 may form a positive feedback loop with cell cycle regulators via the PI3K/AKT/mTOR signaling axis, further promoting the abnormal proliferation of tumor cells. Notably, pro-inflammatory factors secreted by tumor-associated macrophages under hypoxic conditions may synergistically enhance this effect. At the same time, the imbalance in ferroptosis-related pathways may also exacerbate oxidative stress-induced activation of TK1. This multi-dimensional regulatory mechanism provides a new intervention strategy for TK1-targeting tumor therapy.

#### 3.4.5 Challenges and future prospects

As a tumor biomarker, the serum level of TK1 is a reliable indicator of tumor cell proliferation activity and tumor burden, with clear clinical application value. Whether TK1 actively participates in regulating nucleotide metabolism, influences DNA damage repair, and intervenes in key signaling pathways, thereby directly promoting tumor proliferation, survival, invasion, and resistance to chemotherapy (especially antimetabolites) remains unclear. Current research primarily provides correlative evidence. Whether TK1 acts as a core driver oncogene in gastric cancer remains to be confirmed through *in vivo* and *in vitro* functional experiments. Recent studies suggest that TK1 mainly functions as a marker of cell proliferation rather than a direct oncogenic driver. TK1 catalyzes the phosphorylation of thymidine in the DNA salvage synthesis pathway, but there is no direct evidence that it induces malignant transformation (6). Although TK1 is overexpressed in various tumors (such as prostate cancer and lymphoma), and its serum levels are associated with tumor stage and prognosis, no TK1 gene mutations or structural activation directly linked to tumor development have been discovered (53). Research has shown that TK1 knockdown can inhibit tumor cell proliferation; however, this effect is primarily attributed to its inhibition of DNA synthesis rather than to the regulation of oncogenic signaling pathways (54). Therefore, TK1’s tumor-promoting role may be indirect, depending on its biological function in supporting proliferative metabolism (55).

In future research, the specific role of TK1 across different tumor types and its synergistic interactions with other signaling pathways will be important areas of focus. In addition, in-depth exploration of the interaction between TK1 and other key signaling pathways may reveal more potential therapeutic targets, providing a basis for the development of personalized treatment plans.

### 3.5 TK1 as a biomarker: from screening to prognosis

#### 3.5.1 Analytical modalities for TK1 quantification

The STK1 assay demonstrates high specificity in oncological screening, with 87% of STK1-positive individuals exhibiting malignancy-associated pathologies (56). Clinically employed TK1 detection methods encompass radioisotope-based labeling (57), immunoblotting, immunohistochemistry (IHC), chemiluminescent dot blot assays, and enzyme-linked immunosorbent assay (ELISA) (58).

#### 3.5.2 The potential of TK1 as a serum biomarker for gastric cancer

As a biomarker of cell proliferation, TK1 has shown promising potential for diagnosing of gastric cancer in recent years. Recent data suggest that serum levels of TK in digestive tract cancer patients are significantly higher than those in normal people, and significant differences have been observed between patients with and without metastatic tumors (59). Serum TK1 may be elevated in conditions of massive cell proliferation, such as infection, inflammation, and malignancy (42, 60, 61). Therefore, TK level detection is a valuable indicator for tumor diagnosis, tumor malignancy, and prognosis observation. A comparative analysis of serum TK1 levels in patients with gastric ulcers, gastritis, gastric polyps, and gastric cancer revealed significantly elevated TK1 levels in malignant cases compared with healthy controls and benign gastric disease cohorts. Further analysis of the receiver operating characteristic (ROC) curve demonstrated optimal diagnostic performance for gastric cancer at a serum TK1 threshold >95.59 μg/L, yielding sensitivities of 83.3% and 70.0% in distinct validation cohorts. These findings underscore the robust diagnostic potential of serum TK1 as a non-invasive biomarker for discriminating gastric malignancies from non-neoplastic conditions (62).

#### 3.5.3 Phased assessment

Serum TK1 concentration and TK1 expression in tissues by immunohistochemical staining and chemiluminescent dot blotting, respectively, suggesting that TK1 expression is positively correlated with tumor stage and grading (63). Immunohistochemical detection of TK1 can be used to distinguish between benign from malignant tumors. By measuring serum TK1, clinical doctors can detect significant differences in the proliferation of benign and malignant tumor cells (64).

#### 3.5.4 Efficacy monitoring

The study by Liu et al. has demonstrated that changes in TK1 levels during chemotherapy for advanced gastric cancer are closely associated with objective efficacy: a decrease in TK1 after treatment indicates disease remission and longer progression-free survival or relapse-free survival, while an increase in TK1 suggests disease progression. Dynamic monitoring of TK1 changes is more valuable than baseline levels for early prediction of chemotherapy response and survival outcomes in breast cancer (65).

#### 3.5.5 The role of TK1 in prognostic evaluation of gastric cancer prognostic assessment

The expression level of thymidine kinase 1 (TK1) in gastric cancer patients is significantly correlated with their survival. Elevated TK1 levels in the serum of gastric cancer patients are associated with the malignancy, degree of differentiation, and clinical staging of the tumor, as well as poorer postoperative outcomes (24). A study on rectal cancer have reported significantly higher serum TK1 levels in patients with poor prognosis, metastasis, advanced TNM stage, lower differentiation, and lymph node metastasis (66).

#### 3.5.6 Challenges in the clinical translation of TK1

Although TK1 has a sensitivity of 80%(95% CI: 75-85%) and a specificity of more than 90% in human with breast cancer (67), its non-specific elevation is still seen in chronic inflammation, benign tumors, and metabolic diseases (such as fatty liver, gout, etc.), resulting in a high false-positive rate and limiting its application in the diagnosis of a single marker.

Existing detection methods (such as ELISA and CLIA) have fluctuating sensitivity due to differences in reactant affinity (68), and there are differences in TK1 activity in patients with different malignant tumors; for example, the specificity of serum TK1 measured by Western blotting in MDS patients is significantly higher than that in breast cancer and prostate cancer patients (6).

### 3.6 Clinical application prospects and future research directions

TK1 has shown significant clinical potential in tumor diagnosis, prognostic assessment, and treatment monitoring over the past few years. Specifically, it involves pre-treatment testing to identify high-risk patients; regular blood draws during treatment to assess treatment effectiveness or lack of effectiveness earlier than imaging; and follow-up monitoring to detect signs of recurrence in advance. Given the limited specificity of a single biomarker, it will be necessary to combine imaging with other biomarkers (such as CEA and CA19-9) in the future to construct a comprehensive prediction model.

IIn the future, improving detection sensitivity and specificity, as well as the feasibility of clinical applications, should be given greater importance. With the advancement of nanotechnology and molecular biology, developing DNA aptamer-based detection platforms may be a promising direction. For instance, a lateral flow immunochromatographic assay (LFIA) based on a gold nanoparticle-labeled anti-TK1 monoclonal antibody probe can achieve rapid quantitative detection of TK1 with a sensitivity of 0.31 pmol/L, which is highly consistent with the results of chemiluminescent immunoassay kits (69). Besides, a study has shown that the dual monoclonal antibody-based AroCell TK 210 ELISA is a robust, accurate, and precise tool for measuring TK1 protein in various malignancies, thereby improving the clinical applications of TK1 as a biomarker in cancer management (68).

### 3.7 Combined detection of TK1 and other tumor markers

As mentioned above, serum TK1 levels also increase in inflammation and metabolic diseases, potentially leading to false positives. Combining TK1 with other tumor markers for detection can significantly improve the diagnostic accuracy of gastric cancer. Research has shown that combining tK1 detection with markers such as CA724 and DKK1 yielded an area under the receiver operating characteristic (ROC) curve (AUC) of 0.923, which is much higher than the AUC obtained with individual detection. This indicates that combined detection can more effectively identify gastric cancer patients, especially in the early stages (70). Another study showed that TK1 has good specificity (92.0-97.3%), but its sensitivity for diagnosing GC is relatively low (58.1-78.7%). However, when measuring four individual tumor markers (TK1, CA19-9, CA72-4, and CEA), the sensitivity increased to 80.8-88.2% (62).

Using multiple biomarkers together can help comprehensively assess tumor biological characteristics and patient prognosis, thereby achieving the goal of personalized treatment. In the future, the interaction mechanisms between TK1 and these biomarkers should be explored, and the effectiveness of their combined application should be validated through large-scale clinical trials. This will not only help improve early cancer detection rates but may also provide a basis for developing more precise treatment plans for patients.

## 4 Comparison of TK1 functions in gastric cancer and other malignant tumors

### 4.1 Tumor type-specific differences in TK1 expression profiles

The expression of TK1 is not unique to gastric cancer. In other malignant tumors, such as colorectal cancer, serum TK1 levels are also significantly higher than in healthy controls and are associated with tumor stage, histological grade, lymph node status, and metastasis (71). In thyroid cancer, TK1 has been identified as a candidate neoantigen-related gene in early stages, suggesting a specific role in the occurrence and development of thyroid cancer (72). In addition, in breast cancer, high expression of TK1 is associated with more aggressive tumor grading, and its expression levels affect pathogenic pathways such as cell cycle progression, cell migration, and survival (9). These results indicate that TK1 is upregulated in a variety of solid tumors. Still, its expression level and clinical relevance vary significantly across tumor types, reflecting the diversity of tumor biology.

### 4.2 Molecular basis of TK1 functional conservatism and tissue specificity

As a key enzyme in the DNA synthesis salvage pathway, TK1’s core function of catalyzing thymidine phosphorylation is highly conserved evolutionarily. However, in malignant tumors originating from different tissues, the function of TK1 may be regulated by tissue-specific molecular contexts, leading to distinct pathophysiological consequences. In gastric cancer, the function of TK1 is closely related to cell proliferation and chemotherapy response. Studies have shown that sodium butyrate can enhance the sensitivity of gastric cancer cells to 5-fluorouracil (5-FU) by downregulating thymidylate synthase (TS) expression, while also reducing the expression of genes such as TK1 and FOXM1 (73). This suggests that in the mechanism of chemotherapy resistance in gastric cancer, TK1 may have a synergistic or co-regulatory relationship with DNA synthesis-related genes such as *TS*. In breast cancer, TK1 expression directly regulates the transcription of p21 and AKT3, thereby controlling cell cycle arrest, migration, and survival. Its protein interaction network predicts that TK1 has direct or indirect interactions with these pathogenic factors (9). This tissue-specific regulatory network difference may be due to unique activation states of signaling pathways, transcription factor expression profiles, or epigenetic modifications in different tumor cells. For example, the state of *p53* may affect the TK1’s functional output. As seen in gastric cancer cell lines, the effect of sodium butyrate on the cell cycle yields different responses in *p53* wild-type (AGS) and *p53-*deficient (KATO-III) cells (73). Therefore, the upstream regulatory network and downstream effector network of TK1 exhibit tissue-specificity in different malignant tumors, providing the molecular basis for its distinct roles in various cancers.

### 4.3 Differences in the predictive value of TK1 in treatment responses of different malignant tumors

As a marker of cell proliferation, TK1 can vary in its predictive value for response to malignant tumor treatment, depending on the cancer type and treatment strategy. In neoadjuvant chemotherapy of breast cancer, TK1 shows the potential for early prediction of pathological complete remission (pCR). A study constructed a “cell loss” index based on the ratio of serum TK1 concentration to tumor volume and found that changes in this index during early treatment (e.g., 48 hours after the second cycle of chemotherapy) were significantly correlated with the final surgical pathological response. Patients who achieved pCR had a greater increase in cell loss, and this index predicted treatment response with high positive and negative predictive values before the second cycle of treatment (74). This indicates that dynamic changes in TK1 can effectively reflect tumor cell sensitivity to chemotherapy in breast cancer. In gastric cancer, the predictive value of TK1 is reflected in prognostic assessment rather than simply predicting chemotherapy response. Elevated serum TK1 levels are an influencing factor for poor prognosis in gastric cancer patients, and are associated with increased risk of tumor recurrence, metastasis, and death (23). Meanwhile, as mentioned earlier, the downregulation of TK1 expression is associated with the mechanism by which sodium butyrate enhances 5-FU sensitivity, suggesting that it may serve as a potential target or predictive marker for overcoming chemotherapy resistance in gastric cancer (73). In a lymphoma (cat model), serum TK1 activity was significantly increased in diseased animals and returned to normal levels after adequate treatment, indicating its potential for disease diagnosis and treatment monitoring (75).

To sum up, the predictive value of TK1 in the treatment response of different malignant tumors is context dependent: in breast cancer, it may serve as a dynamic monitoring indicator of early efficacy; In gastric cancer, it focuses on the correlation between prognostic stratification and drug resistance mechanisms; In hematological tumors such as lymphoma, it is an effective diagnostic and therapeutic monitoring biomarker. This difference emphasizes the clinical significance of interpreting TK1 based on specific tumor types and treatment backgrounds.

## 5 TK1-targeted therapeutic innovations

In recent years, targeted therapy based on TK1 has been extensively developed. In this review, we mainly divide it into three directions: gene-directed enzyme prodrug therapy (GDEPT), nanotechnology-driven drug delivery, and TK1 inhibitors (**Table 2**).

**Table 2:** TK1-targeted therapeutic strategies and mechanisms.

Therapeutics	Targeting Method	Mechanism of killing cancer cells
Gene-Directed Enzyme Prodrug Therapy (GDEPT)	BF-rTK-GCV	Through gene recombination technology, the thymidine kinase gene is introduced into Bifidobacterium to construct a recombinant strain capable of expressing TK; as well as colonizing and expressing TK in the tumor site, phosphorylate the non-toxic prodrug GCV, and specifically kill tumor cells.
	Adv-TK	Using adenovirus vector to carry the thymidine kinase gene. Adenovirus can infect cells and deliver the *TK* gene into the cells to express TK in the cells.
	*CD/TK* double suicide gene	Expression system contains both *CD* and *TK* double suicide genes. The CD gene can convert 5-fluorocytosine (5-FC) into toxic 5-fluorouracil (5-FU), while the TK gene can phosphorylate ganciclovir (GCV) into a cytotoxic drug.
	AdCEAtk	Using the CEA (carcinoembryonic antigen) promoter, the recombinant adenoviral vector was able to specifically express the herpes simplex virus thymidine kinase (*HSV-tk*) gene in CEA-producing gastric cancer cells.
	*Hsp70B-HSV-tk*/GCV	Highly inducible and tumor-specific promoter activity exists *in vitro* and *in vivo*. HSP-tk/GCV carrying HVJ liposomes combined with hyperthermia can significantly inhibit the growth of subcutaneous tumors and prolong the survival of mice with peritoneal cancer. The combination of suicide gene-directed enzyme prodrug therapy (GDEPT) and hyperthermia provides a promising treatment for advanced gastric cancer.
Nanotechnology-Driven Drug Delivery	PEG-PEI/Fe_3_O_4_	The magnetic field is used to target the tumor site and inhibit the proliferation of BGC823 gastric cancer cells by affecting the intracellular signaling pathway or metabolic process. Thymidine kinase may play a synergistic role in this nanosystem.
	PFH/AGM-CBA/HSV-TK/Liposome (PAHL) -Affibody complex	With ultrasound-targeted microbubble destruction technology and the nuclear localization effect of AGM-CBA vector, the suicide gene containing thymidine kinase gene is transfected into gastric cancer cells. The combined application of multiple technologies improves the efficiency of gene transfection.
	DNA-Integrated Hybrid Nanochannel Sensors	For TK1 activity and inhibition assays. Single-stranded DNA containing thymidine is used as a substrate to functionalize the nanochannel, limiting the ionic current through the channel. The significance of kinase induction can be accurately monitored.
	TK1-mRNA imaging-guided photothermal therapy	The CHA reaction is used to amplify the fluorescent signal, and at the same time, it can generate local high temperature and induce apoptosis of tumor cells
	Logic-gated photodynamic therapy with TK1 in combination with GSH	Activation of near-infrared light-triggered PDT by simultaneously responding to two tumor markers, TK1 mRNA and GSH, through an "AND" logic gate.
TK1 Inhibitors	MKN-74tk + cell	Related to factors such as intracellular signal transduction, gene expression regulation, etc. Providing a basis for selecting appropriate cell models for drug screening and research.
	KDR promoter-driven CDglyTK fusion gene system	Specifically works in cells that highly express KDR (such as SCG7901 and ECV304 cells), inhibiting cell proliferation by expressing components such as thymidine kinase, and exhibits a bystander effect *in vitro*. Killing cells expressing the system without affecting surrounding non-transfected cells.

BF-rTK-GCV, Bifidobacterial recombinant thymidine kinase-ganciclovir; Adv, Adenovirus; CD, Cytosine deoxysidase; CEA, Carcinoembryonic antigen; HSP, heat shock protein; PEG, Polyethylene glycol; PEI, Polyethylenimine; PFH, Perfluorohexane; AGM, agmatine; CBA, N, N'-cystamine-bis-acrylamide; HSV, Herpes simplex virus; CHA, catalytic hairpin assembly; PDT, photodynamic therapy; GSH, glutathione.

### 5.1 Gene-directed enzyme prodrug therapy

#### 5.1.1 Bifidobacterium recombinant thymidine kinase- ganciclovir

By inserting the thymidine kinase (*TK*) gene into Bifidobacterium via genetic recombination, researchers generated a recombinant strain. Once inside the human body, this recombinant strain can colonize tumors and express TK, which phosphorylates the non-toxic prodrug ganciclovir (GCV) into a toxic drug, killing tumor cells specifically. A recombinant Bifidobacterium TK-GCV gene-directed enzyme prodrug therapy (GDEPT) system can effectively induce tumor cell apoptosis, and the mechanism involves activation of the FasL and TNFR2 signaling pathways. It significantly suppresses solid tumor growth without notable toxicity to normal tissues, offering a promising anti-tumor therapeutic strategy and new insights into the treatment of solid tumors (76). Intravenous injection of the BF-rTK/GCV system, mediated by Bifidobacterium and involving herpes simplex virus thymidine kinase, is a safe and effective cancer gene therapy. It elicits a weaker cytokine response than intramuscular injection and inhibits vascular endothelial growth factor (VEGF) expression in mouse models, significantly suppressing gastric tumor growth while ensuring host safety (77). The Bifidobacterium-mediated TK/GCV GDEPT system represents a highly promising and tumor-specific strategy with significant efficacy and an excellent safety profile in preclinical models. Future research should prioritize translational studies, including rigorous toxicology and pharmacokinetic evaluations in larger animal models to pave the way for clinical trials.

#### 5.1.2 Adv-TK

Using advanced adenoviral vector technology to carry the *TK* gene, this system allows adenoviruses to infect cells and deliver the *TK* gene into the cell interior, where it is expressed. The expressed TK phosphorylates nucleoside analogs, selectively killing tumor cells and enhancing the immune response (78). This gene-directed enzyme prodrug therapy (GDEPT) system is highly efficient and targeted.

Immonen et al. Immonen et al. have shown that GDEPT expressed within tumor cells phosphorylates it, generating toxic metabolites that selectively kill tumor cells. This GDEPT system demonstrated highly efficient targeting and anti-tumor activity in clinical trials, confirming its clinical value in the treatment of malignant tumors (79).

Adenovirus-mediated TK/GCV GDEPT represents a highly efficient and targeted therapeutic strategy with confirmed clinical efficacy. Future research should focus on expanding its application to other solid tumors and overcoming the current limitations of adenoviral vectors, particularly their immunogenicity and poor penetration in dense tumor tissues.

#### 5.1.3 Novel transgenic expression system with *CD/TK* dual suicide genes

This system contains *CD* (cytosine deaminase) and *TK* (thymidine kinase) suicide genes. The *CD* gene converts 5-fluorocytidine (5-FC) to toxic 5-fluorouracil (5-FU), while the *TK* gene phosphorylates GCV into a cytotoxic drug. This dual suicide gene system attacks tumor cells from two angles, improving therapeutic efficacy (80–82).

Freytag et al. conducted a phase I clinical trial to evaluate the therapeutic effect of replication-competent adenovirus-mediated dual suicide gene therapy on locally recurrent prostate cancer. The study results showed that the therapy had a favorable safety profile, and tumor volume reduction and disease stabilization were observed in some patients. Although the sample size was limited, the preliminary data suggested that this strategy had potential clinical value in the treatment of refractory prostate cancer, providing a basis for subsequent dose optimization and combination therapy (83).

The CD/TK dual suicide gene system demonstrates enhanced antitumor efficacy through complementary mechanisms of action and a favorable safety profile in early clinical trials. Future research should focus on optimizing viral delivery systems to enhance tumor-specific targeting and transduction efficiency, thereby leveraging potential synergistic effects and addressing tumor heterogeneity.

#### 5.1.4 Recombinant adenovirus vector with CEA promoter

The recombinant adenoviral vector was able to specifically express the herpes simplex virus thymidine kinase (*HSV-tk*) gene in carcinoembryonic antigen (CEA)-producing gastric cancer cells by using the CEA promoter, which can accurately transfer the *HSV-tk* gene to cancerous cells and make them sensitive to GCV, thereby specifically killing gastric cancer cells (84).

Tanaka et al. (85, 86)showed *in vivo* and *ex vivo* experiments that the efficiency of AdCEAlacZ-mediated gene transfer correlated with the amount of CEA produced by each cell line. When tumors contained more than 20% AdCEAtk-infected cells, tumor growth was inhibited after GCV treatment, indicating a practical bystander-killing effect. By intratumoral injection of AdCEAtk, HSVtk was selectively expressed in approximately 30% of CEA-producing cancer cells. By injecting AdCEAtk and GCV administration, tumor growth was significantly inhibited by 20% compared with untreated tumors.

In conclusion, the CEA promoter-driven adenoviral system demonstrates a significant therapeutic effect through both direct and bystander killing mechanisms. Exploring the combination of this targeted gene therapy with conventional chemotherapy or immunotherapy could further improve its clinical efficacy for gastric cancer treatment.

#### 5.1.5 The heat shock protein 70B gene promoter-oriented HSV-tk/ganciclovir system

Isomoto et al. (87) developed a *Hsp70B* gene promoter-directed HSV-tk (HSP-tk)/GCV system. The Luciferase assay suggests highly inducible, tumor-specific promoter activity *in vitro* and *in vivo*. Synergistic effects were also observed when HSV-tk/GCV, mediated by the non-heat-inducible cytomegalovirus (CMV) promoter, and heat treatment were combined, indicating bystander killing. HSP-tk/GCV carrying HVJ liposomes combined with hyperthermia significantly inhibited the growth of subcutaneous tumors in mice with peritoneal carcinoma and prolonged survival. The combination of suicide gene-directed enzyme prodrug therapy (GDEPT) and hyperthermia may provide a promising treatment for advanced gastric cancer. The HSP-tk/GCV system shows an innovative thermo-responsive gene therapy approach that demonstrates significant anti-tumor efficacy through heat-inducible promoter activation. Clinical translation efforts should optimize hyperthermia parameters and delivery systems (e.g., improved liposomal formulations) to maximize therapeutic outcomes for peritoneal carcinomatosis.

### 5.2 Nanotechnology-driven drug delivery

Targeted therapy strategies based on TK1 have gradually become a new direction for the treatment of gastric cancer. The rapid development of nanotechnology has provided an innovative platform for targeted drug delivery, significantly improving drug targeting and therapeutic effects (**Table 3**).

**Table 3:** Nanocarrier efficacy comparison.

Nanosystem	Size range (estimated)	Drug loading/carrier method	*In vivo* tumor inhibition rate (mechanism)	Core technical features
PEG-PEI/Fe3O4	50–200 nm (magnetic NPs)	Chemical bonding/electrostatic adsorption	To be supplemented (magnetic targeting inhibits proliferation via signaling pathways)	Magnetic targeting delivery; synergistic effect of thymidine kinase (TK) enhancement.
PAHL-Affibody Complex	100–500 nm (liposomal)	Gene loading (suicide gene)	To be supplemented (ultrasound-enhanced transfection efficiency)	Ultrasound-targeted microbubble destruction; AGM-CBA nuclear localization; TK gene co-delivery.
DNA-Integrated Nanochannel Sensors	<50 nm (nanochannels)	N/A (detection-focused)	N/A	Single-stranded DNA-functionalized channels; real-time TK1 activity monitoring; drug screening tool.
TK1-mRNA Imaging-Guided PTT	20–80 nm (nanoprobes)	Photothermal agents (e.g., Au NPs)	To be supplemented (apoptosis via localized hyperthermia)	Fluorescence amplification (CHA reaction); TK1-mRNA imaging guidance; photothermal synergy.
Logic-Gated PDT (TK1+GSH)	30–100 nm (responsive)	Photosensitizers (e.g., Ce6)	To be supplemented (dual-marker-activated killing)	"AND" logic gate; simultaneous response to TK1 mRNA and GSH; NIR-triggered with reduced off-target effects.

Size vs. Targeting: Magnetic (PEG-PEI/Fe3O4) and ultrasound-responsive (PAHL) systems favor larger sizes (>100 nm) for external energy-guided targeting.

Photothermal/PDT systems prioritize smaller sizes (<100 nm) for tumor penetration.

Loading Mechanisms: Gene carriers (e.g., PAHL) use plasmid/viral loading.

Photosensitizers/photothermal agents rely on physical encapsulation or covalent conjugation.

Critical Gaps: *In vivo* tumor inhibition rates, quantitative drug loading data, and stability metrics (e.g., blood circulation time) require experimental validation.

#### 5.2.1 Polyethylene glycol-polyethyleneimine/ferroferric oxide (Fe_3_O_4_)

PEG-PEI was used as a carrier and combined with ferroferric oxide Fe_3_O_4_ magnetic nanoparticles to construct a nanostructured system with specific functions. The nanoparticles can target the tumor site via magnetic field effects and may inhibit the proliferation of BGC823 gastric cancer cells by modulating intracellular signal transduction pathways or metabolic processes. Thymidine kinase may play a synergistic role in this nanosystem, such as regulating signal transduction pathways related to cell proliferation (88). This PEG-PEI/Fe3O4-based magnetic nanosystem potentially leverages both physical targeting and biochemical modulation of tumor cell proliferation for targeted therapy. Further investigation is needed to optimize the nanoparticle formulation for enhanced tumor targeting and therapeutic efficacy, thereby maximizing antitumor activity while minimizing systemic toxicity.

#### 5.2.2 PFH/AGM-CBA/HSV-TK/Liposome (PAHL)-affibody complex

Zhou et al. (89) designed a gastric tumor-targeted ultrasound-triggered phase-change nano-ultrasound contrast agent PAHL-Affibody complex. Using ultrasound-targeted microbubble destruction technology and the nuclear localization effect of the AGM-CBA vector, the suicide gene encoding thymidine kinase was transfected into gastric cancer cells. By combining multiple technologies, the efficiency of gene transfection was enhanced, and thymidine kinase played a more effective role in gastric cancer cells, leading to significant apoptosis. The PFH/AGM-CBA/HSV-TK/Liposome-Affibody nanoultrasound contrast agent was developed, offering new ideas for the treatment of ErbB2-positive gastric cancer. The multifunctional ultrasound-triggered nanoplatform achieves enhanced transfection efficiency through the innovative combination of ultrasound-mediated destruction and nuclear-localizing vectors. Future research should focus on optimizing ultrasound parameters and irradiation protocols to maximize gene delivery while minimizing tissue damage.

#### 5.2.3 DNA-integrated hybrid nanochannel sensors

Rauf et al. (90)constructed a hybrid nanochannel sensor integrated with DNA, which was used to detect the activity and inhibition of TK1. Single-stranded DNA containing thymidine was used as a substrate, and functional treatment was used to limit the ion current in the nanochannel. Upon kinase action, the thymidine residues at the end of the substrate DNA were phosphorylated, increasing transmembrane ion current, thereby increasing surface charge density and alleviating pore blockage. The hybrid nanodevice can accurately monitor kinase-mediated phosphorylation reactions and demonstrates efficient, specific evaluation in human serum samples. This nanochannel-based biosensor offers a highly sensitive and specific platform for monitoring phosphorylation events in complex biological samples. Future research should focus on validating this detection platform in larger clinical cohorts to establish its diagnostic utility for early cancer detection and monitoring.

#### 5.2.4 TK1-mRNA imaging-guided photothermal therapy

A polydopamine-based nucleic acid nanoprobe (PDA-DNA) has been developed to enable precise imaging and treatment of tumor cells by targeting TK1 mRNA (91). The probe uses a catalytic hairpin assembly (CHA) reaction to amplify the fluorescent signal with a detection limit as low as 9.34 pM, which is two orders of magnitude more sensitive than traditional non-amplified probes. At the same time, under 808 nm laser irradiation, the nanoprobe can generate local high temperatures and induce tumor cell apoptosis, thereby achieving synergistic imaging and photothermal therapy (91). PDA-DNA achieves exceptional sensitivity in TK1 mRNA detection while integrating precise imaging with photothermal treatment capabilities. Further development should aim to optimize the photothermal conversion efficiency and targeting specificity, explore combination therapies with chemotherapeutic agents or immunotherapies to enhance antitumor effects.

#### 5.2.5 Logic-gated photodynamic therapy with TK1 in combination with GSH

Another study designed intelligent nanomachines (D/UCNMs) to simultaneously respond to two tumor markers, TK1 mRNA and glutathione (GSH), via an “AND” logic gate, thereby activating near-infrared light-triggered photodynamic therapy (PDT). The system uses specific recognition of TK1 mRNA, combined with enzyme-free amplification technology, to significantly improve the spatiotemporal accuracy of treatment and to monitor drug distribution in real time via NIR-II fluorescence imaging (92). This “AND” logic gate-controlled nanoplatform achieves unprecedented specificity through dual-marker recognition while integrating real-time monitoring with targeted photodynamic therapy. Future research should focus on validating this sophisticated system *in vivo* to evaluate its targeting efficiency and therapeutic outcomes in complex tumor microenvironments.

### 5.3 Overcoming chemoresistance: TK1 inhibitors

#### 5.3.1 MKN-74tk + cells

Azatian A et al. (93) found that the herpes simplex virus thymidine kinase gene (HSV-tk) can be a suicide gene when combined with the precursor drug ganciclovir (GCV). Introduction of the HSV thymidine kinase gene using an HSV vector, followed by GCV administration, resulted in significant cell death *in vitro* and tumor regression *in vivo*. Comparison of the sensitivity of different cell lines, human gastroesophageal junction adenocarcinoma cell line SK-GT-515, and human gastric adenocarcinoma cell line MKN-74 to ganciclovir (GCV). It is suggested that thymidine kinase responds differently to drugs in different cellular contexts, which may be related to intracellular signaling and gene expression regulation. This comparative study may provide a basis for selecting suitable cell models for drug screening and research. The study confirms the therapeutic potential of HSV-tk/GCV suicide gene therapy while highlighting the critical importance of cellular context in determining treatment response. Future research should focus on elucidating the precise molecular mechanisms underlying these cell-type-specific differences, particularly the role of intracellular signaling pathways and gene expression networks that modulate TK-mediated drug sensitivity.

#### 5.3.2 KDR promoter-driven CDglyTK fusion gene system

Qiang Li et al. (94) investigated the inhibitory effect of the KDR promoter-driven adenovirus-mediated fusion gene system on the proliferation of human gastric adenocarcinoma SCG7901 cells *in vitro*. The CDglyTK fusion gene system driven by the KDR promoter could specifically function in cells with high KDR expression, such as SCG7901 and ECV304 cells. The fusion gene system inhibits cell proliferation by expressing components such as thymidine kinase. It exhibits a bystander effect *in vitro*, i.e., it not only kills cells expressing the system but also inhibits the surrounding untransfected cells. The KDR promoter-driven CDglyTK fusion gene system demonstrates excellent tumor-specific targeting and potent anti-tumor efficacy through dual suicide gene expression. Future research should focus on optimizing the transcriptional targeting strategy by exploring tumor-specific promoters with even greater specificity and activity to create synergistic anti-tumor effects that target both tumor cells and their vascular microenvironment.

#### 5.3.3 Targeted therapeutic mechanisms against the cell cycle

Targeted therapeutic strategies against thymidine kinase 1 (TK1) are transforming cancer treatment by disrupting its functional network. In gene-directed enzyme prodrug therapy (GDEPT), recombinant adenoviral vectors such as AdCEAtk deliver exogenous TK1 genes into gastric cancer cells, leading to specific overexpression. This process converts the non-toxic prodrug GCV into its active form, GCV-TP, which damages DNA and triggers apoptosis.

Additionally, the toxic metabolite spreads to neighboring tumor cells through intercellular gap junctions, expanding the treatment’s reach. Meanwhile, PEG-PEI/Fe3O4 nano-delivery systems target tumor tissues with magnetic precision, releasing TK1 inhibitors that block dTMP synthesis. This blockage disrupts the nucleotide pool, halting S-phase DNA replication and curtailing cell proliferation.

Furthermore, TK1 inhibitors, such as GCV analogues, competitively bind to TK1’s active site, stifling its function and leading to dTMP depletion. Together, these strategies not only target TK1’s enzymatic activity but also initiate a multifaceted attack against tumors by modulating various signaling networks, marking a significant advance in oncology.

Future research should focus on developing next-generation TK1-targeting platforms with enhanced tumor specificity and reduced off-target effects, and conducting comprehensive translational studies to bridge the gap between preclinical efficacy demonstrations and clinical application in gastric and other TK1-overexpressing cancers.

## 6 Challenges and future perspectives

TK1 is a key enzyme in the pyrimidine nucleotide salvage pathway, and its serum level has been widely shown to be closely associated with the occurrence and progression of gastric cancer. It is also regarded as a critical biomarker for the diagnosis and prognosis of gastric cancer. Recent evidence highlights the clinical utility of serum TK1 quantification in early gastric cancer screening, clinical staging, monitoring treatment efficacy, and prognostic assessment. In recent years, the role of TK1 in the drug resistance of gastric cancer has gradually attracted public attention. Studies have confirmed that it mediates resistance to 5-FU in gastric cancer cells by regulating cell cycle and metabolic pathways. In addition, epidemiological surveys have shown that TK1 expression is significantly positively correlated with H. pylori infection and the severity of precancerous lesions in gastric mucosal epithelial tissue. New targeted therapeutic strategies based on TK1’s molecular characteristics, including gene therapy, cytotoxic killing, and nanotechnology-driven drug delivery, have shown promising clinical trial results.

However, the specific molecular mechanism of TK1 in the occurrence and development of gastric cancer has not yet been fully elucidated, which, to some extent, limits the development of gastric cancer prevention strategies based on TK1 (95). Despite the significant potential of TK1 in diagnosing and treating gastric cancer, its clinical translation faces several challenges. The elevated non-specificity of serum TK1 results in a high rate of false positives, caused by conditions such as chronic inflammation and metabolic diseases, which limits its effectiveness for independent use. Existing assays also exhibit poor comparability across different platforms due to variations in antibody affinity and inadequate operational standardization. Furthermore, the immunogenicity of adenoviral vectors used in gene therapies, such as Gene-Directed Enzyme Prodrug Therapy (GDEPT), can trigger host-neutralizing antibody responses, thereby diminishing tumor-targeting efficiency. Additionally, long-term use of TK1 inhibitors, like Ganciclovir (GCV), may lead to resistance through metabolic reprogramming or the activation of alternative signaling pathways.

Notably, although it has been proposed that TK1 may play a role in Hp’s antibiotic resistance, this remains speculative and lacks direct experimental evidence. In the future, detailed mechanistic studies, such as using gene-knockout and overexpression models with multi-omics analysis, are needed to systematically assess the specific contribution of TK1 resistance to various antibiotics in both *in vivo* and *in vitro* environments. At the same time, the impact of different Hp strains’ genetic backgrounds and variations in the infection microenvironment on TK1 function expression should be considered to avoid one-sided conclusions.

Future research can focus on the following directions: first, in-depth analysis of the signal transduction network mediated by TK1 in the occurrence and development of gastric cancer to provide a theoretical basis for the development of new targeted immunotherapy drugs; second, explore the synergistic mechanism of TK1 inhibitors and anti-Hp treatment and develop combined treatment plans; finally, use new technologies such as single-cell sequencing and spatial transcriptomics to systematically clarify the interaction mechanism between TK1 and the gastric cancer microenvironment. Ongoing research on the molecular mechanism of TK1 will provide new insights into early prevention and precision treatment of gastric cancer and is expected to improve the clinical prognosis of gastric cancer patients significantly.
